# Comparison of Suturing Techniques on Wound Healing and Patient-Centered Outcomes After Non-impacted Tooth Extraction: An Observational Study

**DOI:** 10.7759/cureus.110820

**Published:** 2026-06-14

**Authors:** M.G. Dharmendra Kumar, S.Y. Rajan, Shilpa Sunil Khanna, Deepak Kolte, Dipayan Datta, Prathyusha Vemuri, Abdul Ahad Ghaffar Khan, Rahul V.C. Tiwari

**Affiliations:** 1 Department of Oral and Maxillofacial Surgery, CKS Theja Institute of Dental Sciences and Research, Tirupati, IND; 2 Department of Oral Medicine and Radiology, Vananchal Dental College and Hospital, Garhwa, IND; 3 Department of Oral and Maxillofacial Surgery, Sri Ramakrishna Dental College and Hospital, Coimbatore, IND; 4 Department of Oral and Maxillofacial Surgery, Bharati Vidyapeeth (Deemed to be University) Dental College and Hospital, Navi Mumbai, IND; 5 Department of Public Health Dentistry, Agartala Government Dental College, Agartala, IND; 6 Department of Prosthodontics and Crown and Bridge, Care Dental College, Guntur, IND; 7 Department of Oral and Maxillofacial Surgery, College of Dentistry, King Khalid University, Abha, SAU; 8 Department of Oral and Maxillofacial Surgery, RKDF Dental College and Research Centre, Bhopal, IND

**Keywords:** oral surgery, postoperative pain, suture technique, tooth extraction, wound healing

## Abstract

Introduction: Successful wound closure after tooth extraction is important for postoperative healing and patient comfort. Different suturing techniques may influence tissue approximation, wound stability, postoperative inflammation, and patient-centered outcomes. The present study aimed to compare the effects of simple interrupted suturing (SIS), figure-of-eight suturing (F8S), and horizontal mattress suturing (HMS) on postoperative wound healing and patient-related clinical outcomes following routine non-impacted tooth extraction.

Materials and methods: This prospective observational cohort study included 150 participants requiring routine non-impacted tooth extraction, with 50 participants enrolled in each suturing group: SIS, F8S, and HMS. Group assignment was based on surgeon preference and patient choice. Soft tissue healing was assessed using the early wound healing score on postoperative days 1, 3, 7, and 14. Secondary outcomes included postoperative pain (assessed using the visual analog scale), edema, postoperative bleeding, wound dehiscence, suture handling time, and patient satisfaction. All statistical analyses were performed using IBM SPSS Statistics for Windows, Version 23 (Released 2016; IBM Corp., Armonk, New York, United States), with a significance level set at p < 0.05.

Results: Baseline demographic and clinical characteristics were comparable between the groups (p > 0.05). Early wound healing scores progressively improved over time in all groups. However, the HMS group had the highest healing scores, followed by the SIS group. On postoperative day 14, healing scores were 9.7 ± 0.4 for HMS, 9.6 ± 0.5 for SIS, and 8.9 ± 0.8 for F8S. On postoperative day 1, pain scores were highest in the F8S group (5.82 ± 1.41) compared with the SIS (4.25 ± 1.23) and HMS (3.98 ± 1.15) groups. Edema, postoperative bleeding, and wound dehiscence were also significantly greater in the F8S group (p < 0.05). Although F8S required the shortest handling time (52.1 ± 9.7 seconds), patient satisfaction was significantly lower compared with the other groups.

Conclusion: Within the limitations of this study, SIS and HMS demonstrated superior postoperative healing and patient-centered outcomes compared with F8S following routine tooth extraction. HMS showed the most favorable healing profile, whereas F8S, despite requiring shorter operative time, was associated with inferior postoperative outcomes.

## Introduction

Tooth extraction is one of the most frequently performed procedures in oral and maxillofacial surgery, and successful postoperative healing is essential to minimize complications, reduce patient discomfort, and restore oral function [1]. Although extraction sockets generally heal uneventfully, the quality of soft tissue approximation and stabilization immediately after extraction plays a critical role in early wound healing, postoperative pain control, hemostasis, and prevention of wound dehiscence [2]. Therefore, suturing techniques are considered important determinants of postoperative recovery and patient satisfaction following dental extraction [3].

Various suturing methods have been described in oral surgery, including simple interrupted suturing (SIS), figure-of-eight suturing (F8S), and horizontal mattress suturing (HMS) [4]. SIS is widely used because of its ease of placement, precise wound edge approximation, and adaptability to different extraction sites [4]. F8S is commonly employed to provide compression over the extraction socket and promote stable blood clot formation, particularly when hemostasis is a concern [5]. HMS offers broader tissue engagement and enhanced wound margin eversion, potentially improving tissue stability and vascular perfusion during healing [6]. Despite their routine clinical use, there is limited evidence regarding the comparative effectiveness of these techniques on postoperative healing and patient-reported outcomes of routine extraction procedures.

Previous studies have primarily focused on specific surgical scenarios, such as third molar surgery or periodontal procedures, while evidence concerning routine non-impacted tooth extraction remains insufficient [3,7]. Furthermore, differences in postoperative pain, edema, bleeding, wound dehiscence, and patient satisfaction among the commonly used suturing techniques have not been comprehensively evaluated in real-world observational settings. Understanding these differences may help clinicians to select the most appropriate suturing approach for improved healing and patient comfort.

Therefore, the present study aimed to compare the effects of SIS, F8S, and HMS on postoperative soft tissue healing, pain intensity, edema, bleeding, wound dehiscence, suture handling time, and patient satisfaction following routine non-impacted tooth extraction.

## Materials and methods

Study design and setting

This prospective observational cohort study was conducted at the Department of Oral and Maxillofacial Surgery of the RKDF Dental College and Research Centre, Bhopal, India, from December 2018 to November 2019. The study protocol was approved by the Institutional Ethics Committee (RKDF/DC/IEC/2018/SS-25), and all procedures were performed in accordance with the ethical principles of the Declaration of Helsinki. The study was designed and reported according to the Strengthening the Reporting of Observational Studies in Epidemiology (STROBE) guidelines [8]. Written informed consent was obtained from all participants prior to their enrollment in the study.

Participant selection

Systemically healthy adult patients aged between 18 and 65 years requiring extraction of a single non-impacted tooth, excluding the third molars, were consecutively screened for eligibility. The teeth indicated for extraction included those with severe caries, periodontal disease, root stumps, or non-restorable conditions. Only patients with adjacent teeth and relatively healthy surrounding periodontal tissues were included to ensure uniformity in the healing assessment. Patients with acute odontogenic infections or abscesses at the extraction site; uncontrolled systemic diseases; pregnancy or lactation; history of head and neck radiotherapy, bisphosphonate therapy, immunosuppressive therapy, chronic corticosteroid use; heavy smoking habits (>10 cigarettes/day); allergy to local anesthetics or suture materials; or those requiring flap elevation, bone removal, or grafting procedures were excluded from the study. Patients who were unable to comply with the follow-up visits were also excluded.

Sample size determination

A total of 150 participants were included in the study, with 50 allocated to each suturing technique group. The sample size was determined based on feasibility and a previously published study demonstrating clinically meaningful differences in soft tissue healing scores of approximately 1.2 points with a standard deviation of 1.5 [9]. A post hoc power analysis was performed using G*Power software version 3.1.9.7 (Heinrich Heine University Düsseldorf, Düsseldorf, Germany), which demonstrated that a sample size of 50 participants per group provided 85% statistical power at an alpha level of 0.05 to detect significant pairwise differences between groups.

Group assignment

The study was conducted as an observational study without randomization. The assignment to the suturing groups was based on surgeon preference and patient choice, after a detailed explanation of the available suturing techniques. Two experienced oral surgeons participated in the study, and each surgeon routinely performed the preferred suturing techniques in clinical practice (SSK and DK). Because group allocation was based on surgeon preference and patient choice, the distribution of cases across techniques was not identical among surgeons. Surgeon identity was therefore recorded and included as a covariate in multivariable analyses to minimize operator-related bias. After tooth extraction, patients were informed about the characteristics of each suturing method, and the final selection was documented. The participants were categorized into three groups: Group A, SIS; Group B, F8S; and Group C, HMS, with 50 participants in each group.

Surgical procedure and suturing techniques

All extraction procedures were performed under local anesthesia using 2% lignocaine with 1:80,000 adrenaline injections (LOX 2% Adrenaline, Neon Laboratories Ltd., Mumbai, Maharashtra, India). Standard atraumatic extraction techniques using periotomes and elevators were used whenever possible. Following tooth extraction, the socket was irrigated with sterile normal saline solution, and hemostasis was achieved with sterile gauze pressure.

The suturing procedures were performed using 4-0 non-resorbable monofilament polyamide sutures (Ethilon®; Ethicon Inc., Somerville, New Jersey, USA). In the SIS group, two to three interrupted sutures were placed, approximating the buccal and lingual/palatal gingival margins over the extraction socket. In the F8S group, a single crossed suture was placed over the extraction socket to stabilize the blood clot and to provide compression. HMS were placed in the HMS group, engaging the buccal and lingual gingiva approximately 3-4 mm away from the wound margins to provide broad tissue stabilization [3-6].

Postoperative instructions were standardized for all participants. Patients were instructed to avoid rinsing or spitting for 24 hours, consume a soft diet for three days, and carefully maintain oral hygiene. Chlorhexidine gluconate 0.12% mouthwash (Hexidine®, ICPA Health Products Ltd., Mumbai, Maharashtra, India) was prescribed twice daily from the second postoperative day for 14 days. Ibuprofen 400 mg tablets (Brufen®, Abbott Laboratories, Chicago, Illinois, USA) were prescribed as rescue analgesics when required. The sutures were removed on the seventh postoperative day by an assistant who was not involved in outcome evaluation.

Outcome assessment

Clinical outcomes were evaluated at baseline and postoperatively on days 1, 3, 7, and 14 by a calibrated examiner blinded to the suturing technique used (RT). Calibration exercises were performed before commencement of the study to ensure examiner reliability. The primary outcome of the study was early soft tissue healing assessed using the early wound healing score (EHS) described by Marini et al. [10] (which is an open-access article distributed under the terms and conditions of the Creative Commons Attribution (CC BY) license). EHS consists of three parameters: clinical signs of re-epithelialization, clinical signs of hemostasis, and clinical signs of inflammation. Re-epithelialization was scored as 0, 3, or 6, while hemostasis and inflammation were scored as 0, 1, or 2. The cumulative score ranged from 0 to 10, with higher scores representing superior healing. Independent scores from both examiners were averaged for statistical analysis. Inter-examiner and intra-examiner reliabilities were assessed using weighted kappa statistics with a target agreement value greater than 0.80.

Postoperative pain was assessed using a freely available 10-cm visual analog scale (VAS), where 0 represented no pain, and 10 represented the worst imaginable pain [11]. Participants recorded their pain intensity daily for seven days in a standardized diary. Postoperative bleeding was assessed at one, three, and 24 hours following extraction and recorded as either present or absent, depending on the occurrence of active bleeding or oozing requiring intervention. Facial edema was evaluated using a standardized three-point facial measurement method involving the tragus-pogonion, tragus-commissure, and outer canthus-gonion distances [12]. The difference between the baseline and postoperative measurements on day 3 represents the severity of edema.

Wound dehiscence was clinically evaluated on postoperative days 3, 7, and 14 and categorized as present or absent depending on the separation of the wound margins. Suture handling time was recorded in seconds from initial needle insertion to completion of the final knot using a digital stopwatch by an independent assistant. Patient satisfaction was assessed on day 30 using a 5-point Likert scale ranging from 1 (very dissatisfied) to 5 (very satisfied).

Data collection and follow-up

All clinical findings and patient-reported outcomes were documented by an independent research assistant (AAGK). Participants were followed up throughout the postoperative period, and any adverse events, such as dry socket, infection, persistent bleeding, or allergic reactions, were recorded and managed according to institutional protocols. In cases of missed follow-up visits, participants were contacted telephonically and encouraged to attend subsequent appointments. Available data from participants who were lost to follow-up until their last visit were included.

Statistical analysis

Statistical analysis was performed using IBM SPSS Statistics for Windows, Version 23 (Released 2016; IBM Corp., Armonk, New York, United States). Data normality was assessed using the Shapiro-Wilk test. Continuous variables were expressed as mean ± standard deviation or median (interquartile range), as appropriate, while categorical variables were presented as frequencies and percentages. Baseline demographic and clinical characteristics among the three suturing groups were compared using one-way analysis of variance (ANOVA), Kruskal-Wallis test, or chi-square test, as appropriate. Potential confounders were selected a priori based on clinical relevance and previous literature and included age, sex, body mass index, smoking status, tooth type, tooth location, extraction difficulty, surgeon identity, and estimated intraoperative blood loss.

The primary outcome, EHS, was analyzed using a linear mixed-effects model with suturing technique group, time interval, and group-by-time interaction as fixed effects and patient identity as a random effect. Age, tooth location, extraction difficulty, and surgeon identity were included as covariates. Bonferroni correction was applied for pairwise comparisons. Secondary outcomes, including postoperative pain, edema, and suture handling time, were analyzed using analysis of covariance (ANCOVA). Postoperative bleeding and wound dehiscence were analyzed using the chi-square test, while patient satisfaction was evaluated using ordinal logistic regression. Statistical significance was set at p < 0.05, and all tests were two-tailed.

## Results

A total of 150 participants were included in the study, with 50 allocated to each suturing group (SIS, F8S, and HMS). All participants completed follow-up assessments and were included in the final analysis.

Baseline demographic and clinical characteristics

No statistically significant differences were observed between the three groups in terms of age, sex distribution, body mass index, smoking status, tooth location, tooth type, extraction difficulty, or intraoperative bleeding (all p > 0.05). These findings indicated that the groups were comparable at baseline, allowing for a reliable comparison of postoperative outcomes among the suturing techniques (Table 1).

**Table 1:** Baseline demographic and clinical characteristics of the three suturing groups. Data were presented as mean ± standard deviation, median (interquartile range), or number (percentage); one-way analysis of variance (ANOVA) was used for normally distributed continuous variables; the Kruskal-Wallis test was used for non-normally distributed variables; the chi-square test was used for categorical variables; p < 0.05 was considered statistically significant. SIS: simple interrupted suturing, F8S: figure-of-eight suturing, HMS: horizontal mattress suturing, BMI: body mass index, SD: standard deviation, IQR: interquartile range

Characteristic	SIS (n = 50)	F8S (n = 50)	HMS (n = 50)	Test statistic	p‑value
Age (years), mean ± SD	42.3 ± 12.1	44.1 ± 11.8	41.9 ± 13.4	F = 0.482	0.618
Sex, female, n (%)	28 (56)	26 (52)	30 (60)	χ^2^ = 0.640	0.726
BMI (kg/m^2^), mean ± SD	24.5 ± 3.2	24.9 ± 3.5	24.2 ± 3.1	F = 0.548	0.579
Smoking (≤10/day), n (%)	10 (20)	12 (24)	9 (18)	χ^2^ = 0.556	0.757
Tooth location - mandible, n (%)	32 (64)	35 (70)	30 (60)	χ^2^ = 1.099	0.577
Tooth type - posterior, n (%)	38 (76)	41 (82)	36 (72)	χ^2^ = 1.476	0.478
Extraction difficulty, n (%)					
Easy	30 (60)	28 (56)	32 (64)	χ^2^ = 1.440	0.837
Moderate	16 (32)	18 (36)	14 (28)		
Complex	4 (8)	4 (8)	4 (8)		
Intraoperative bleeding (mL), median (IQR)	5.0 (3.0-8.0)	5.5 (3.5-9.0)	4.5 (3.0-7.5)	H = 1.842	0.398

EHS over time

All groups demonstrated progressive improvements in healing scores from day 1 to 14. The HMS group consistently showed the highest healing scores, followed closely by the SIS group, whereas the F8S group showed comparatively lower scores at all the time points. Linear mixed-effects analysis revealed a significant effect of suturing technique, time, and group × time interaction on wound-healing outcomes (p < 0.001). These findings suggest that healing progression differed significantly among the three suturing techniques during the postoperative period (Table 2).

**Table 2:** Early wound healing score over time among the three suturing groups. Data were presented as mean ± standard deviation; a linear mixed-effects model was used for repeated-measures analysis with group, time, and group × time interaction as fixed effects; *p < 0.05 was considered statistically significant. SIS: simple interrupted suturing, F8S: figure-of-eight suturing, HMS: horizontal mattress suturing, SD: standard deviation

Interaction	Time point	SIS (n = 50)	F8S (n = 50)	HMS (n = 50)	F	p‑value	Effect size (Partial η^2^)
Group	Day 1	5.2 ± 1.3	4.8 ± 1.2	5.4 ± 1.4	8.24	<0.001*	0.101
	Day 3	6.9 ± 1.1	6.0 ± 1.3	7.1 ± 1.2			
	Day 7	8.5 ± 0.9	7.6 ± 1.1	8.7 ± 0.8			
	Day 14	9.6 ± 0.5	8.9 ± 0.8	9.7 ± 0.4			
Time	Day 1	5.2 ± 1.3	4.8 ± 1.2	5.4 ± 1.4	342.1	<0.001*	0.699
	Day 3	6.9 ± 1.1	6.0 ± 1.3	7.1 ± 1.2			
	Day 7	8.5 ± 0.9	7.6 ± 1.1	8.7 ± 0.8			
	Day 14	9.6 ± 0.5	8.9 ± 0.8	9.7 ± 0.4			
Group X Time	Day 1	5.2 ± 1.3	4.8 ± 1.2	5.4 ± 1.4	3.56	<0.001*	0.046
	Day 3	6.9 ± 1.1	6.0 ± 1.3	7.1 ± 1.2			
	Day 7	8.5 ± 0.9	7.6 ± 1.1	8.7 ± 0.8			
	Day 14	9.6 ± 0.5	8.9 ± 0.8	9.7 ± 0.4			

Pairwise comparison of healing scores

On day 1, the HMS group demonstrated significantly higher EHS scores than the F8S group (p = 0.014), whereas the difference between the SIS and F8S groups was not statistically significant. On days 3 and 7, both SIS and HMS groups exhibited significantly better healing than the F8S group (all adjusted p < 0.01). No significant differences were observed between SIS and HMS at any postoperative interval. By day 14, intergroup differences were no longer statistically significant (Table 3).

**Table 3:** Pairwise comparisons of early wound healing scores among the suturing groups at different postoperative time points. Pairwise comparisons were performed using Bonferroni correction following linear mixed-effects model analysis; adjusted p-values were reported; *p < 0.05 was considered statistically significant. SIS: simple interrupted suturing, F8S: figure-of-eight suturing, HMS: horizontal mattress suturing

Comparison	Day	t	Adjusted p	Cohen’s d	95% CI for d
SIS vs. F8S	1	2.15	0.096	0.43	(0.03, 0.83)
	3	3.42	0.004*	0.68	(0.28, 1.09)
	7	3.52	0.002*	0.70	(0.30, 1.11)
	14	1.18	0.480	0.24	(-0.16, 0.63)
HMS vs. F8S	1	2.84	0.014*	0.57	(0.17, 0.97)
	3	4.05	<0.001*	0.81	(0.40, 1.22)
	7	4.18	<0.001*	0.84	(0.43, 1.24)
	14	0.95	0.612	0.19	(-0.20, 0.58)
SIS vs. HMS	1	0.63	0.796	0.13	(-0.27, 0.52)
	3	0.58	0.812	0.12	(-0.28, 0.51)
	7	0.76	0.449	0.15	(-0.24, 0.55)
	14	0.32	0.875	0.06	(-0.33, 0.46)

Secondary outcomes

Postoperative pain scores were significantly higher in the F8S group at all evaluated time points, whereas the HMS group reported the lowest pain intensity. Significant intergroup differences in the pain scores were observed on days 1, 3, and 7 (all p ≤ 0.002). Suture handling time varied significantly among the groups (p < 0.001). The F8S technique required the shortest operative time, whereas HMS required the longest time for placement. Postoperative edema was the highest in the F8S group and the lowest in the HMS group, with statistically significant intergroup differences (p < 0.001). Bleeding and wound dehiscence were observed more frequently in the F8S group than in the SIS and HMS groups. Significant differences were noted in postoperative bleeding (p = 0.033) and wound dehiscence (p = 0.024) (Table 4).

**Table 4:** Comparison of secondary postoperative outcomes among the three suturing groups. Data were presented as mean ± standard deviation or number (percentage); analysis of covariance (ANCOVA) was used for postoperative pain, edema, and suture handling time; chi-square test was used for bleeding and wound dehiscence; *p < 0.05 was considered statistically significant. SIS: simple interrupted suturing, F8S: figure-of-eight suturing, HMS: horizontal mattress suturing, VAS: visual analog scale, SD: standard deviation **Eta square for continuous variables and Cramer V for categorical variables.

Parameters	Metric	SIS (n = 50)	F8S (n = 50)	HMS (n = 50)	Test values	p‑value	Effect size**
Postoperative pain day 1	Mean ± SD	4.25 ± 1.23	5.82 ± 1.41	3.98 ± 1.15	F = 9.14	<0.001*	0.11
Postoperative pain day 3	Mean ± SD	2.84 ± 0.92	4.15 ± 1.18	2.59 ± 0.87	F = 8.77	<0.001*	0.11
Postoperative pain day 7	Mean ± SD	1.23 ± 0.51	1.98 ± 0.74	1.15 ± 0.49	F = 6.42	0.002*	0.08
Suture handling time (in seconds)	Mean ± SD	68.5 ± 12.3	52.1 ± 9.7	94.3 ± 15.8	F = 121.4	<0.001*	0.62
Edema (in mm from baseline to day 3)	Mean ± SD	4.2 ± 1.8	6.1 ± 2.2	3.9 ± 1.6	F = 12.6	<0.001*	0.15
Bleeding present	N (%)	5 (10)	12 (24)	4 (8)	χ^2^ = 6.82	0.033*	0.21
Dehiscence present	N (%)	2 (4)	8 (16)	1 (2)	χ^2^ = 7.44	0.024*	0.22

Patient satisfaction

The highest proportion of satisfied and very satisfied patients was observed in the HMS group, followed by the SIS group. In contrast, the F8S group demonstrated lower satisfaction levels, with a greater proportion of dissatisfied responses (Table 5).

**Table 5:** Patient satisfaction scores at postoperative day 30 among the three suturing groups. Data were presented as numbers (percentages); patient satisfaction was assessed using a 5-point Likert scale. SIS: simple interrupted suturing, F8S: figure-of-eight suturing, HMS: horizontal mattress suturing

Score	SIS (n = 50)	F8S (n = 50)	HMS (n = 50)
1 (very dissatisfied)	0 (0.0%)	2 (4.0%)	0 (0.0%)
2 (dissatisfied)	1 (2.0%)	8 (16.0%)	1 (2.0%)
3 (neutral)	4 (8.0%)	12 (24.0%)	3 (6.0%)
4 (satisfied)	20 (40.0%)	18 (36.0%)	22 (44.0%)
5 (very satisfied)	25 (50.0%)	10 (20.0%)	24 (48.0%)

The ordinal logistic regression analysis (Table 6) demonstrated that patients treated with the F8S technique had significantly lower odds of achieving higher satisfaction scores than those treated with the SIS technique (OR = 0.32, p < 0.001). No significant difference in satisfaction was observed between the HMS and SIS groups (p = 0.813).

**Table 6:** Ordinal logistic regression analysis for patient satisfaction among the suturing groups. An ordinal logistic regression model was used to evaluate the likelihood of higher patient satisfaction scores; SIS was used as the reference category; *p < 0.05 was considered statistically significant. OR: odds ratio, CI: confidence interval, SIS: simple interrupted suturing, F8S: figure-of-eight suturing, HMS: horizontal mattress suturing

Comparison	OR (95% CI) for higher satisfaction	z value	p‑value
F8S vs. SIS	0.32 (0.16-0.64)	-3.42	<0.001*
HMS vs. SIS	1.08 (0.56-2.08)	0.24	0.813

## Discussion

This prospective observational study comparatively evaluated the influence of SIS, F8S, and HMS on postoperative wound healing and patient-centered outcomes following routine tooth extraction. The findings demonstrated that SIS and HMS were associated with significantly superior early wound healing, lower postoperative pain, reduced edema, lower incidence of bleeding and wound dehiscence, and greater patient satisfaction than F8S. However, F8S required significantly less suturing time than other techniques. This observation is clinically understandable because F8S closure generally involves placement of a single crossed suture over the socket, making the procedure technically faster and less demanding than techniques requiring multiple suture passes.

Early soft tissue healing is an important determinant of postoperative recovery after oral surgical procedures, because appropriate wound stabilization promotes re-epithelialization, clot protection, and reduction of postoperative inflammatory complications [2]. In this study, the HMS group demonstrated the highest EHS values, followed closely by the SIS group, whereas the F8S group showed consistently lower healing scores during the first postoperative week. The significant group × time interaction further indicated that the healing trajectory differed among suturing techniques. These findings may be attributed to the broader tissue support and tension distribution achieved by HMS, which reduce wound edge ischemia and improve tissue approximation [13]. Similarly, SIS permit precise adaptation of wound margins while minimizing excessive tissue compression [9,13]. In contrast, F8S mainly provide socket compression rather than ideal marginal adaptation, potentially compromising vascular perfusion and delaying soft tissue healing [14].

The present findings are consistent with those of the study by Koyuncu and Gürses [9], who reported significantly better wound healing with continuous suturing than with interrupted sutures during oral surgical wound closure. They emphasized that the suturing technique itself may influence healing independent of the suture material. Previous studies have also suggested that adequate wound edge approximation, reduction of dead space, and prevention of alveolar osteitis are critical factors for favorable healing in oral surgery [15]. Furthermore, mattress sutures have been reported to improve flap stability and vascularity by distributing tension over broader tissue surface [13,16].

Postoperative pain was significantly greater in the F8S group at all the evaluated time intervals. This finding may be explained by the increased compression exerted by the crossed suture over the extraction socket, resulting in greater tissue tension and local inflammatory response. This finding was in contrast to previous studies, where F8S was found to be effective in reducing postoperative pain [5,17]. This difference might be due to the fact that these studies were done on deeply impacted third molars. Excessive tissue strangulation may impair local blood circulation and contribute to the prolonged release of inflammatory mediators, thereby increasing pain perception [18]. In contrast, the HMS and SIS demonstrated lower pain scores owing to more physiological tissue approximation and improved wound stability. Similar observations were reported by Zhao et al. [14], who evaluated F8S following third molar surgery and noted that although the technique aided clot stabilization, it did not substantially improve postoperative pain outcomes.

The present study also demonstrated significantly greater postoperative edema in the F8S group. Swelling after oral surgery is primarily associated with tissue trauma and inflammatory exudation. Suturing techniques that produce excessive compression may compromise microcirculation and lymphatic drainage, resulting in increased edema formation [18]. HMS distributes pressure more evenly across tissues and may, therefore, minimize localized tissue trauma [9]. Additionally, better wound stability may reduce inflammatory edema during the early postoperative period. These observations support the findings of previous oral surgery studies that emphasized the relationship between flap tension, tissue perfusion, and postoperative swelling [16,19].

Bleeding and wound dehiscence were significantly more common in the F8S group than in the SIS and HMS groups. Although F8S are frequently used to compress extraction sockets and control bleeding, excessive localized pressure may paradoxically compromise clot stability and impair marginal tissue adaptation [14]. Inadequate adaptation of wound edges may also predispose patients to partial wound separation during functional oral movements. In contrast, interrupted and mattress sutures provide a more stable approximation of wound margins, thereby reducing the risk of postoperative dehiscence [5,6]. Previous investigations evaluating oral surgical sutures have similarly highlighted the importance of tension-free wound closure in preventing soft tissue separation and delayed healing [3,16].

An important finding of the present study was that F8S required the shortest suturing time, whereas HMS required the longest. This observation is clinically understandable because F8S generally involves placement of a single crossed suture over the socket, making the procedure technically faster and less demanding [14]. HMS, on the other hand, requires multiple needle passes and more precise tissue engagement [6]. Similar findings were reported by Koyuncu and Gürses [9], who observed a shorter suturing time with continuous suturing techniques than interrupted sutures. Although reduced operative time may improve procedural efficiency, the present results suggest that faster closure does not necessarily translate into superior postoperative healing outcomes.

The patient satisfaction findings further support the clinical superiority of the SIS and HMS techniques. Both groups demonstrated substantially higher satisfaction scores than the F8S group, and ordinal logistic regression analysis confirmed the significantly lower odds of higher satisfaction in patients treated with F8S. Patient satisfaction after oral surgery is multifactorial and influenced by pain, swelling, wound healing, and postoperative comfort [20]. Therefore, the inferior satisfaction observed with the F8S may reflect the greater pain, edema, bleeding, and dehiscence associated with this technique. In contrast, improved tissue healing and reduced postoperative morbidity in the SIS and HMS groups likely contributed to the better patient-reported experiences.

Although SIS and HMS demonstrated superior healing during the early postoperative period, pairwise comparisons showed that intergroup differences were no longer statistically significant by postoperative day 14. Therefore, the principal clinical advantage of these techniques appears to relate to accelerated early healing and reduced postoperative morbidity rather than sustained long-term differences in soft tissue healing. This finding suggests that the choice of suturing technique may be particularly important during the initial postoperative period when patient discomfort and wound complications are most likely to occur.

Clinical implications and limitations

The findings of the present study suggest that SIS and HMS techniques were associated with superior early postoperative healing, reduced postoperative morbidity, and improved patient-centered outcomes compared with F8S following routine tooth extraction. The observed benefits were most evident during the initial healing phase, indicating that suturing technique may play an important role in promoting early wound stability, minimizing discomfort, and enhancing patient recovery. Clinicians should therefore consider wound stability and tissue adaptation, in addition to operative convenience, when selecting suturing techniques for routine oral surgical procedures.

However, several limitations should be considered when interpreting these findings. The observational, non-randomized design may have introduced selection bias despite statistical adjustment for measured confounding variables. Furthermore, allocation based on surgeon preference and patient choice may have contributed to residual confounding, and the influence of unmeasured factors cannot be completely excluded. Although surgeon identity was included as a covariate in the multivariable analyses, residual operator-related effects may still have influenced the outcomes. In addition, patient satisfaction was assessed at postoperative day 30, and participants were aware of the suturing technique selected as part of the observational study design. This may have introduced expectation bias, potentially affecting satisfaction scores independent of actual clinical outcomes. Importantly, while SIS and HMS demonstrated superior healing during the early postoperative period, healing scores became comparable among the groups by postoperative day 14, suggesting that the principal advantage of these techniques relates to accelerated early healing rather than persistent long-term differences in soft tissue healing. The study was also conducted at a single center with a relatively short follow-up period, which may limit the generalizability of the findings. Future multicenter randomized controlled trials with larger sample sizes, blinded outcome assessment, and longer follow-up periods are warranted to further validate these findings.

## Conclusions

Within the limitations of the present study, SIS and HMS techniques demonstrated superior early wound healing, reduced postoperative pain and edema, lower incidence of bleeding and wound dehiscence, and higher patient satisfaction compared with F8S following routine tooth extraction. However, healing scores became comparable among the groups by postoperative day 14. Although F8S required less operative time, SIS and HMS were associated with more favorable early postoperative outcomes and may therefore be preferred when enhanced early healing and patient comfort are clinical priorities.
